# Female Blow Flies As Vertebrate Resource Indicators

**DOI:** 10.1038/s41598-019-46758-9

**Published:** 2019-07-22

**Authors:** Charity G. Owings, Aniruddha Banerjee, Travis M. D. Asher, William P. Gilhooly, Anais Tuceryan, Mary Huffine, Christine L. Skaggs, Iyun M. Adebowale, Nicholas E. Manicke, Christine J. Picard

**Affiliations:** 1 Department of Biology, Indiana University-Purdue University Indianapolis, 723 W Michigan St, SL 306, Indianapolis, IN 46202 USA; 2 Department of Geography, Indiana University-Purdue University Indianapolis, 425 University Blvd, Cavanaugh Hall 441, Indianapolis, IN 46202 USA; 3 Department of Earth Sciences, Indiana University-Purdue University Indianapolis, 723 W Michigan St, SL 118, Indianapolis, IN 46202 USA; 4 International School of Indiana, 4330 N Michigan Rd, Indianapolis, IN 46208 USA; 5 Department of Chemistry and Chemical Biology, Indiana University-Purdue University Indianapolis, 402 N Blackford, LD 326, Indianapolis, IN 46202 USA

**Keywords:** Biodiversity, Food webs

## Abstract

Rapid vertebrate diversity evaluation is invaluable for monitoring changing ecosystems worldwide. Wild blow flies naturally recover DNA and chemical signatures from animal carcasses and feces. We demonstrate the power of blow flies as biodiversity monitors through sampling of flies in three environments with varying human influences: Indianapolis, IN and two national parks (the Great Smoky Mountains and Yellowstone). Dissected fly guts underwent vertebrate DNA sequencing (12S and 16S rRNA genes) and fecal metabolite screening. Integrated Nested Laplace Approximation (INLA) was used to determine the most important abiotic factor influencing fly-derived vertebrate richness. In 720 min total sampling time, 28 vertebrate species were identified, with 42% of flies containing vertebrate resources: 23% DNA, 5% feces, and 14% contained both. The species of blow fly used was not important for vertebrate DNA recovery, however the use of female flies versus male flies directly influenced DNA detection. Temperature was statistically relevant across environments in maximizing vertebrate detection (mean = 0.098, sd = 0.048). This method will empower ecologists to test vertebrate community ecology theories previously out of reach due practical challenges associated with traditional sampling.

## Introduction

Biodiversity is integral to ecosystem health and stability, and the loss thereof can have dramatic and cascading consequences on a global scale^1–3^. Given this, it is imperative to implement quick, non-invasive methods to evaluate spatiotemporal changes in animal community compositions^4^. However, traditional surveillance techniques are labor-intensive^5–7^, and modern minimal-effort techniques have disadvantages. For example, camera trapping requires sensitive image processing, large data capacities, and may carry a body-size bias^8–10^. Additionally, such methods limit the number of taxa or guilds that can be evaluated simultaneously, requiring implementation of several methods (increasing sampling effort and cost) for total community assessment.

Recent studies suggest utilizing invertebrates, such as blow flies (Diptera: Calliphoridae), to indirectly monitor vertebrates^11–13^, with evidence that flies out-perform traditional survey methods in detecting animal richness^14,15^. Many blow fly species are necrophagous, requiring an animal carcass as a larval developmental substrate^16^. In order to select quality resources to lay eggs, adult female blow flies “taste” carcasses in the wild, effectively sampling and storing host DNA in their bodies. Additionally, female flies also visit animal feces for highly desirable protein, which initiates development of their reproductive organs^17^. As host DNA can also be recovered from epithelial cells found on the exterior surface of feces^18^, flies can pick up vertebrate DNA in addition to fecal metabolites when feeding on this type of resource. Given this unique biology of the blow fly, vertebrate DNA (now contained within the fly) can be extracted and sequenced^14,19^ and combined with an analytical assay for fecal metabolites^20^, constituting a powerful approach for remote species identifications in any environment with conditions supporting blow fly activity.

The black blow fly *Phormia regina* (Meigen, Diptera: Calliphoridae) was the primary blow fly species of interest in this study. This species is highly abundant and one of the most forensically important blow flies in the United States^21,22^. The overarching goals of this work were to enhance current blow fly-based vertebrate DNA methods and illustrate the depth of environmental information gleaned from flies collected from different ecosystems.

## Results

### Effect of subsampling

**Figure 1:**
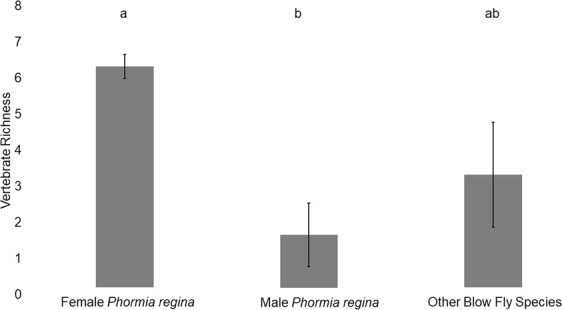
Bar plots summarizing the mean vertebrate richness (with mean standard error bars) detected from blow flies from the subsampling experiment. Statistically different values are represented with different letters (P < 0.05).

Entire samples (i.e. both sexes of all species) of blow flies were collected at three urban parks (Military Park, Skiles Test Park, Province Park; Table 1) in and around Indianapolis, IN, USA and analyzed to determine the ideal fly species, sex, and sample size for recovering vertebrate diversity. ANOVA revealed a significant difference among the sexes of flies sampled for vertebrate DNA (*P* = 0.043), with a post-hoc test showing that female *P*. *regina* detected significantly more vertebrate species than males (*P* = 0.038, Fig. 1). 33% of female *P*. *regina* tested positive for vertebrate DNA, compared to 21% and 14% in male *P*. *regina* and blow flies of other species, respectively. No statistically significant differences were detected between either female or male *P*. *regina* and blow flies of other species. There was a significant difference in richness when sample sizes increased from 10 to 15 or 20 flies (*P* < 0.001), though there was no difference between 15 and 20 flies. Therefore, up to 15 flies per sample should recover maximum vertebrate richness.

**Table 1:** Summary of geographic regions and sites used for blow fly collections.

Region	Site	City, State	Coordinates	Flies Analyzed
Urban (March–Oct. 2016)	^#^Military Park	Indianapolis, IN	39.770555, −86.168611	20 (30.3%); ^#^53 (100%)
	Northwest Park	Greenwood, IN	39.628611, −86.143611	30 (30.9%)
	^#^Skiles Test Park	Indianapolis, IN	39.867882, −86.048541	20 (12.3%); ^#^69 (100%)
	University Park	Greenwood, IN	39.611061, −86.050641	30 (28.3%)
	^#^Province Park	Franklin, IN	39.477500, −86.053333	^#^141 (100%)
Smokies (11–13 June 2018)	Site 1	near Gatlinburg, TN	35.734722, −83.413333	20 (13.6%)
	Site 2	near Gatlinburg, TN	35.704444, −83.364722	20 (13.0%)
	Site 3	near Gatlinburg, TN	35.663330, −83.526389	26 (17.1%)
	Site 4	near Gatlinburg, TN	35.670833, −83.680000	30 (5.2%)
Yellowstone (9–11 July 2018)	Site 1	near Gardiner, MT	44.614170, −110.413600	26 (39.4%)
	Site 2	near Gardiner, MT	44.957780, −110.541700	30 (22.6%)
	Site 3	near Gardiner, MT	44.957780, −110.311900	22 (32.4%)
	Site 4	near Gardiner, MT	44.885560, −110.144400	20 (37.7%)

Dates given reflect sampling periods. Urban = Indianapolis, IN, USA, Smokies = Great Smoky Mountains national park, Yellowstone = Yellowstone national park. Except for the subsampling experiment (indicated by a^#^) which examined both sexes of all blow fly species present, only female P. regina were selected for analyses in all other samples. The number of flies analyzed per site, as well as the percentage of flies analyzed out of the entire sample (in parentheses), is given in the last column.

*Number and percentage of flies analyzed for site.

### Wild sampling

Active sampling (240 min/region) yielded a total of 1,784 *P*. *regina*, of which 293 females were analyzed. Overall, 28 vertebrate species were identified, with 42% of flies containing vertebrate resources: 23% DNA, 5% feces, and 14% both. Not all fecal positive flies resulted in a positive species identification. Resource-seeking behavior is likely influenced by the biology of the female fly, with gravid females (i.e. females with fully developed eggs) more likely to sample vertebrate resources. This was indeed observed in the data as slightly more than half (53%) of the analyzed flies were gravid, and of those gravid females, 64% contained vertebrate animal information (37% vertebrate DNA, 21% fecal metabolites, and 6% both).

#### Urban

**Figure 2:**
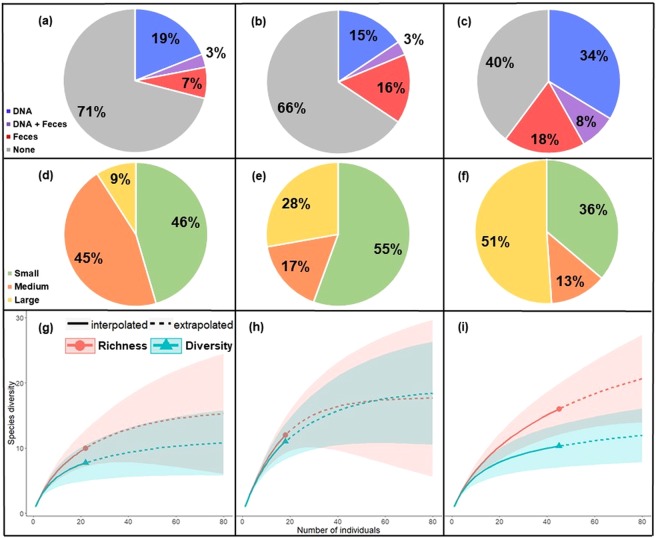
Summary of vertebrate abundance and diversity detected by blow flies from three regions: urban (left column), Smokies (middle column), and Yellowstone (right column). (**a**–**c**) Pie charts showing the abundance (given as percentages) of flies containing evidence of vertebrate resources (DNA, feces, and flies containing both vertebrate DNA and feces), as well as flies with no vertebrate resources detected. (**d**–**f**) Pie graphs showing relative body sizes (small, medium, large) of vertebrate species detected by flies. (**g**–**i**) Rarefaction curves generated from vertebrate data showing both interpolated and extrapolated values for vertebrate richness and diversity (shaded areas represent 95% confidence intervals).

Of the 434 *P*. *regina* collected in the urban environment, 23% were analyzed. 29% of these flies contained vertebrate resources: 19% DNA, 7% feces, and 3% both (Fig. 2a). Most sequenced animal species (>50%) were small to medium (S = 10; Fig. 2d,g and Table 2). Rarefaction and extrapolation of species richness and diversity determined that a plateau is approached when sampling ~40 flies (Fig. 2g). Unsurprisingly, dogs (*Canis lupus familaris*) were the most common animal detected in the urban environment (making up 37% of all positive species IDs).

**Table 2:** Summary of vertebrate species detected by blow flies in three regions: urban, Smokies, and Yellowstone.

Species	Urban	Smokies	Yellowstone
*Canis lupus familiaris*	7	2	0
*Sylvilagus floridanus*	3	2	4
*Felis catus*	2	1	1
*Sus scrofa*	1	1	0
*Bos taurus*	1	2	0
*Marmota monax*	1	0	0
*Peromyscus leucopus*	1	0	0
*Procyon lotor*	3	0	0
*Sciurus niger*	2	1	0
*Didelphis virginiana*	1	0	0
*Odocoileus sp*.	0	1	13
*Sciurus carolinensis*	0	3	0
*Ursus americanus*	0	1	1
*Canis latrans*	0	1	0
*Cavia porcellus*	0	2	0
*Neovison vison*	0	1	0
*Peromyscus sp*.	0	0	1
*Cervus elaphus*	0	0	8
*Myodes sp*.	0	0	2
*Mus sp*.	0	0	1
*Taxidea taxus*	0	0	1
*Vulpes vulpes*	0	0	1
*Bison bison*	0	0	2
*Felis sp*.	0	0	1
*Antilocapra americana*	0	0	2
*Cynomys sp*.	0	0	3
*Urocitellus armatus*	0	0	3
*Canis sp*.	0	0	1

The lowest taxonomic level resolved by the primers used in this study is given for each animal.

#### National parks

The abundance of *P*. *regina* collected from the Smokies and Yellowstone was 1030 (9% analyzed) and 320 (31% analyzed), respectively. Though only 36% of Smokies flies contained vertebrate resources (15% DNA, 16% feces, 3% both; Fig. 2b), most (>50%) were small mammals (S = 12, Fig. 2e,h and Table 2). In contrast, 68% of Yellowstone flies contained vertebrate resources (34% DNA, 18% feces, 8% both), with >50% being large-bodied animals (S = 15; Fig. 2f,i and Table 2). Rarefaction and extrapolation of richness and diversity shows that Yellowstone requires more sampling to reach a plateau compared to the Smokies (Fig. 2h,i). 11% of DNA-positive Smokies flies contained dog DNA, whereas only one Yellowstone fly detected a single occurrence of *Canis lupus*, though whether this was a dog or a wolf could not be resolved given the presence of large wolf packs in Yellowstone and the limitations on domestic dogs in the park. Therefore, in an effort to be conservative, the authors only report the genus of this animal (Table 2). Additionally, the uncommon Pine Marten (*Martes americana*), and a previously unobserved prairie dog genus (*Cynomys* sp.) were detected in Yellowstone (Table 2)^23^. However, as 4 of the 23 rodent species in subfamily Xerinae documented within the park have not been sequenced at the loci used for this study, it is not known whether the *Cynomys* sp. detected here is a true representation of this genus in the park, or whether it is the result of an incomplete molecular database.

### Parameters affecting vertebrate resource detection

**Figure 3:**
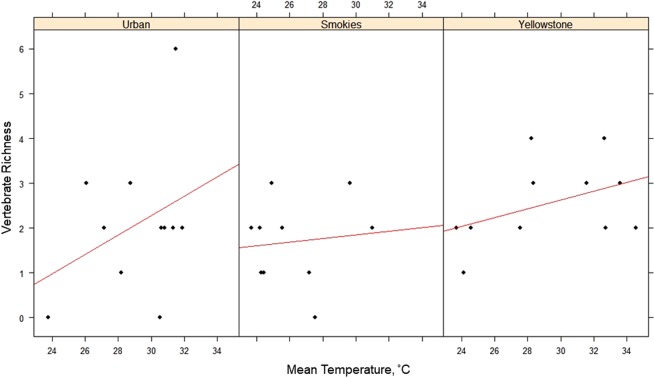
Scatterplots of vertebrate richness by mean temperature for three regions: Urban, Smokies, and Yellowstone. Regression lines are shown in red.

Hierarchical Bayesian inference using INLA showed no significant results for predicting vertebrate DNA- or fecal-positivity in flies. However, a positive interaction between mean temperature and vertebrate richness was observed (mean = 0.098, sd = 0.048, 0.025Q = 0.004, 0.975Q = 0.192; Fig. 3). Other variables (humidity, wind speed, abundance of gravid flies, and total abundance of flies) had no impact on vertebrate detection by flies in the areas and timeframes in which flies were sampled. A chi-square test for independence determined that vertebrate DNA detection and fecal metabolite detection were statistically independent of one another (χ^2^ = 3.35, df = 2, *P* = 0.187).

## Discussion

Study design is crucial for successfully surveying vertebrate biodiversity with invertebrates. We show that by analyzing a fraction of collected flies (average = 21%) in a limited timeframe (240 min/environment), 26–43% of common and abundant mammals (excluding bats) can be detected in three distinct environments^23–25^. Vertebrate DNA detected by flies approximated the mammal richness in the sampled regions. Using vertebrate-specific primers producing short (<250 bp) amplicons, we were able to detect the same number of wild animal species in our urban samples (S = 6, excluding four domestic animal species) as a recent study in Germany, though without the use of blocking primers or next generation sequencing^13^. Similarly, richness values, as well as vertebrate detection rates, from flies captured at the national parks were comparable to previous studies investigating carrion DNA from flies in pristine tropical habitats^11,14,15^.

Though it is not surprising that higher temperatures on collections days resulted in more vertebrate species being detected, this is important to point out for non-entomologists wishing to use insects as vertebrate diversity indicators. The poikilothermic nature of insects leads them to be more active during warm weather^26^, increasing the speed of population turnover and quickly leading to increases in population abundance. For blow flies, this translates to increased active searching behaviors for required resources, thereby increasing the vertebrate diversity detected by flies. Though the other abiotic variables tested here seemed to have very little impact on vertebrate diversity detected by flies, additional sampling (especially on a temporal scale) would likely reveal more significant microclimatic effects.

Additionally, we have shown that wild female blow flies are better candidates for vertebrate DNA recovery compared to males, likely due to their nutritional requirements for reproduction. While males will visit vertebrate resources such as feces for their own sexual maturation^17^, the frequency at which they do so appears to be much less than females. Implementing the use of female blow flies in practice is quite easy to do, even for the non-entomologist. The most obvious sexually dimorphic characteristic to use is the placement of the eyes: the eyes of males are proportionally large and touch in the middle of the head, while the eyes of females are proportionally smaller and separated, usually by an appreciable distance^27^. This simple delineation can be easily used by researchers to enhance the recovery of vertebrate DNA in sampled wild flies.

We have also illustrated the utility of actively capturing blow flies in a limited timeframe and analyzing only female blow flies individually via gut dissection. Active sampling is ideal in situations where repeated sampling in an area is not possible, as well as because it is known exactly when flies arrive at the bait and when they are killed by the researcher. Furthermore, the measurement of abiotic factors, like wind speed and direction, during active sampling could potentially be important in determining where the fly may be traveling from, as blow flies will fly into the odor plume when attempting to locate the bait. Passive trapping does have its benefits as it may require much less effort on the part of the researcher and potentially result in larger collections over time^14^. However, there is always a chance that baited passive traps can be scavenged or destroyed by other animals. If implemented for iDNA studies, passive traps should be checked frequently to avoid degradation of DNA and potential disturbance by other animals. In terms of preparation of flies, some studies pool samples prior to molecular diet analysis^13,15^. Though this method may be more cost-efficient when performing next generation sequencing to quickly asses biodiversity of an area, it has been shown that pooling can reduce the number of detectable vertebrate species in a sample^14^. This is likely due to “rare” or low template DNA within individual flies (potentially due to low acquisition or decay of DNA over time) being lost due to PCR bias, resulting in an underestimate of vertebrate diversity. Additionally, valuable individual data, such as whether or not the fly has fed on feces as well as the reproductive status of the fly, is lost if samples are pooled. Overall, the risks and rewards of fly sampling and molecular analysis methods should be weighed carefully against the overall goal of the study.

Our results also show that the process resulting in the presence of vertebrate DNA in flies is potentially independent from the process resulting in the presence of fecal metabolites. This could mean that either (1) the acquisition of these materials occurs separately (i.e. the fly visited both a fecal resource, where it picked up fecal metabolites, and a carcass, where it picked up DNA), or, (2) if both DNA and feces were acquired together, one material must degrade more rapidly than the other. One previous report found that vertebrate DNA from beef liver tissue was detected in blow fly guts up to 96 h after ingestion by the blow fly *Chrysomya megacephala* (Macquart)^19^. On the other hand, we have found that fecal metabolites can persist in *P*. *regina* for as long as two weeks post-ingestion (Supplementary Fig. 1, Supplementary Data). The sensitivity of the chemical fecal assay^20^ in conjunction with the persistence of the urobilinoid signal in the fly guts points to the high reliability of this assay to detect fecal metabolites if they have been ingested by the fly. Given this finding, it would make more sense that if DNA and fecal metabolites were ingested concurrently, that the fecal signal would outlast the DNA signal over time since feeding. In the context of our wild fly sampling data, this would mean that the flies containing only a vertebrate DNA signal likely obtained the DNA from a carcass or carrion resource. Though feces can serve as an adequate protein source for flies and they seem to visit it frequently^16^, the most preferable resource for females is animal carrion. Female blow flies in various stages of vitellogenesis will visit carrion or carcasses^28–30^, and protein from this type of resource results in much more rapid egg maturation^17^. As for the flies containing both DNA and feces, it cannot be determined whether DNA was obtained from the fecal resource alone or if two different resources were ingested.

It is important to acknowledge the dispersal potential of blow flies in order to determine if fly-derived data represents the true vertebrate community composition of the area of interest. A comprehensive review of blow fly dispersal outlines the variability in distances traveled by flies, albeit this likely results from a combination of variable abiotic factors (i.e. wind speed, temperature, overall climate) during sampling, as well as varying recovery methods utilized by researchers^16^. Local spatial aggregation is well-known in blow flies, suggesting that though individual flies in a population may disperse somewhat randomly, the population itself persists in an area due to both resource availability and environmental factors^31–33^. However, it has also been reported that flies can travel up to 13 km in 24 h likely in search of important nutrients or resources which may be lacking in the area^34^. In the context of vertebrate resource sampling, female flies (the flies most important for the method presented in this paper) likely would not disperse far away from areas rich in protein (e.g. vertebrate feces) and oviposition resources (e.g. animal carcasses on which to lay their eggs). Thus, vertebrate DNA detected from female flies should reflect the true vertebrate diversity present in a relatively local area of collection. If sampling in a geographic location that is known to have low vertebrate diversity (e.g. large agricultural areas with little to no animal refugia), then caution should be taken in interpreting vertebrate data detected by flies as the region in which these animals may actually be located likely lies outside of the local area of fly collection.

Overall, blow flies contain vast reservoirs of environmental information waiting to be tapped by scientists. Additional ecological data can also be extracted from flies, such as population genetics of targeted species^12^, vertebrate-pathogen associations^35,36^, and even angiosperm diversity in phenological studies (wild blow flies consume nectar^37,38^ and potentially pollen)^39^. Furthermore, flies can be used to monitor environmental pollution (e.g. pesticides, agricultural run-off) using similar analytical chemistry methods used to detect fecal metabolites. The possibilities of using blow flies as environmental monitors are nearly endless. With the tools provided here, it is possible to detect taxa spanning multiple trophic levels and spatiotemporal scales with a limited sampling effort, an endeavor unattainable using any other current methods.

## Methods

### Blow fly collections

#### Fly sampling

**Figure 4:**
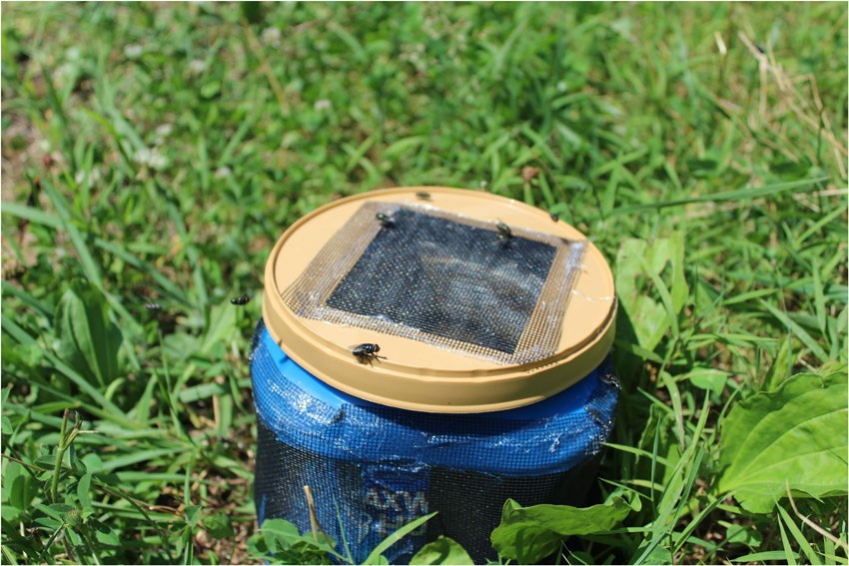
Holding container for aged chicken liver bait used to attract blow flies. The sides of the container, as well as the lid, have been cut open and covered with mesh. This aerates the container allowing for attractive volatile cues to disseminate into the surrounding environment while preventing the flies from landing on the bait itself.

Blow flies were sampled three times from four urban sites (Indianapolis, IN, USA) and four sites in two national parks: Great Smoky Mountains National Park (= Smokies) and Yellowstone National Park (=Yellowstone; Table 1, (Permits: GRSM-2018-SCI-2039; YELL-2018-SCI-7046). A decayed chicken liver bait inside an aerated container was used to attract flies during each 20 min sampling period (Fig. 4). Flies were collected with an aerial sweep net and killed in 70% ethanol on site.

#### Subsampling

Three urban public parks were investigated at a single timepoint to determine the ideal blow fly species and sample size for vertebrate surveillance (Table 1).

### Molecular and chemical analysis

For subsampling experiments, both sexes of all blow fly species collected from each park were analyzed (Military Park: 53 flies; Skiles Test Park: 69 flies; Province Park: 131 flies). For all other sampling, the black blow fly, *Phormia regina* Meigen (Diptera: Calliphoridae), was analyzed due to its prevalence in the USA. A maximum of 10 female *P*. *regina* were randomly selected from each spatiotemporal sample for analysis.

#### Dissections and DNA extractions

Crops and hindguts of each fly were removed using flame-sterilized forceps and placed inside a sterile 1.5 mL microcentrifuge tube. Flies containing mature eggs were deemed gravid. Dissection of guts was followed by digestion in 200 uL ChargeSwitch® lysis buffer (Invitrogen™, Carlsbad, CA, USA) and 10 uL 20 mg/mL proteinase K (Invitrogen™), incubating for 4 h at 60 °C. 100 uL phenol chloroform isoamyl alcohol (PCI, 25:24:1) (Thermo Fisher™, Waltham, MA, USA) was added to each lysate and centrifuged at 5,000 rpm for 5 min, separating the extraction into an organic “waste” layer and an aqueous DNA layer. A standard phenol-chloroform DNA extraction was continued^40^.

#### Chemical analysis

Waste layers of each sample underwent vertebrate fecal metabolite analysis according to methods previously outlined^20^.

#### Molecular vertebrate species identification

Several vertebrate-specific primer pairs used for amplifying vertebrate DNA in similar studies were tested during preliminary controlled feeding experiments with flies (data not shown). These included two different cytochrome b primer pairs (UNFOR403/UNREV1025^41^ and L14841/H15149)^42^, two 12S rRNA primer pairs (12SV5F/12SV5R^43^ and L1085/H1259)^44^, and two 16S rRNA primer pairs (16SMam1/16SMam2^45^ and L2513/H2714)^44^. Both sets of cytochrome b primers resulted in the amplification of fly DNA, which obscured any vertebrate data that may have been obtained. Success rate was variable with the 12S V5 primers as well as the 16SMam primers. The optimal primers for this study were determined to be the 12S (L1085/H1259; 215 bp amplicon) and 16S (L2513/H2714; 244 bp amplicon) rRNA mitochondrial primers as they consistently produced successful amplification and sequencing of vertebrate DNA without amplifying fly DNA^44^. For each 10 uL total volume PCR reaction, each of the following were added: 5 uL Promega 2X PCR mastermix (Promega™, Madison, WI, USA), 1 uL 5 uM forward and reverse primers, 0.5 uL 1X bovine serum albumin (BSA; Promega™), and 2.5 uL genomic DNA. Amplifications were carried out on a Mastercycler Pro thermocycler (Eppendorf^®^, Hamburg, Germany) using a 10-step touchdown from 63 °C to 54 °C, then 25 cycles at 54 °C, and a final extension at 7 min.

Samples were purified with 1 uL ExoSAP-IT™ (ThermoFisher Scientific, Waltham, MA, USA) following manufacturer’s protocols. Amplicon sequencing was performed according to BigDye™ Terminator v3.1 Cycle Sequencing kit (ThermoFisher) protocols. Sequencing products were purified via ethanol precipitation: 1.25 uL 125 mM EDTA was dissolved into each sample, followed by 20 uL 100% ethanol, and incubation for 15 min at room temperature. A second centrifugation was conducted at 2500 g for 30 min, followed by removal of ethanol. 20 uL 70% ethanol was added and the plate was centrifuged at 2500 g for 15 min. After ethanol removal, the plate was inverted and centrifuged at 185 g for 1 min. Samples were re-suspended in 10 uL HiDi™ formamide (ThermoFisher), vortexed for 15 s, and denatured at 95 °C for 5 min. Sequences were separated and detected on a 3500 genetic analyzer (ThermoFisher) and manually edited and trimmed via Sequence Scanner (ThermoFisher). Only sequences with clearly resolved nucleotides were used (low quality or obvious multiple sequences were discarded). Sequences were then queried using the National Center for Biotechnology Information (NCBI) nucleotide database using BLASTn®. Only the top hit with a query coverage of >95% and an e-value < 10^−5^ was accepted.

### Vertebrate diversity analyses

Reference databases for DNA sequences (like NCBI GenBank) are not complete, which can be a limitation in iDNA studies performed in areas where many native vertebrate species are not represented in the database. Additional assignment methods have been shown to enhance BLAST results (MEGAN) or outperform them altogether (PROTAX)^14^. As the environments sampled in this study had well-characterized vertebrate taxa that were represented in GenBank at the loci used for amplification, the top BLAST result was used with confidence as the true species identification. However, in the event that an appropriate match or identification was not made, a phylogenetic analysis was performed to infer the lowest-possible taxonomic level of samples that could not be resolved to species (i.e. the phylogenetic analyses were used to resolve to either genus or family level). Sequences of multiple genera based on animal distributions were downloaded from NCBI, sequence alignments were done in MEGA-X v10.0.4^46^, and a Tamura-Nei distance tree (500 bootstrap replicates) was generated. Animal body sizes (kg) were obtained from Quaardvark^47^ and placed into three subjective categories: small (<5 kg), medium (5–55 kg), and large (>55 kg). Vertebrate species richness (S) and the extrapolation of richness from rarefaction curves were obtained using the R packages *vegan*^48^ and *iNEXT*^49^, respectively.

### Statistical analyses

For subsampling experiments, a one-way ANOVA with a post-hoc Tukey’s honest squared differences was performed with native R packages^50^ to elucidate differences in richness among female *P*. *regina*, male *P*. *regina*, and all other blow flies analyzed. To determine the ideal fly sample size needed to maximize richness, subsamples of N = 10, 15, and 20 flies were randomly generated from the three subsampling datasets and analyzed via Kruskal-Wallis test with post-hoc Dunn’s test using *dunn*.*test*^51^.

An Integrated Nested Laplace Approximation (INLA) algorithm^52^ specifically implementing the Besag, York, and Mollie (BYM) spatial autoregression^53^ using Hierarchical Bayesian Inference (HBI) was used to determine the most important predictors of vertebrate resource availability. Conditional autoregressive (CAR, random walk) Bayesian Hierarchical Model captures the hierarchical nature of space (both correlated and uncorrelated spatial heterogeneity) and time (both time trend and space-time diffusion) and incorporates abiotic factors as predictors. These predictors included mean temperature (°C), mean humidity (%RH), mean wind speed (m/s), abundance of *P*. *regina*, and abundance of gravid females per sample. Response variables included abundance of vertebrate DNA-positive flies, vertebrate richness, and abundance of feces-positive flies per sample. A chi-squared test for independence was also conducted to assess whether the detection of vertebrate DNA and fecal metabolites were independent of each other.

## Supplementary information

Supplementary Figure 1

Supplementary Dataset 1

## Data Availability

Data is archived via OSF: https://osf.io/2bvdn.
