# Supplementary material for: Female Blow Flies As Vertebrate Resource Indicators
Source: Sci Rep. 2019 Jul 22;9:10594. doi: 10.1038/s41598-019-46758-9 (PMC6646386; doi:10.1038/s41598-019-46758-9)
Supplement: Supplementary file 1 — Supplementary Figure 1 [file 41598_2019_46758_MOESM1_ESM.pdf]

Supplementary Information

FEMALE BLOW FLIES AS VERTEBRATE RESOURCE INDICATORS

*Charity G. Owings[1], Aniruddha Banerjee[2], Travis M. D. Asher[2], William P. Gilhooly III[3], Anais Tuceryan[4], Mary Huffine[1], Christine H. Skaggs[5], Iyun M. Adebowale[5], Nicholas E. Manicke[5], Christine J. Picard[1]

Supplementary Figure 1. Line graph showing the proportion of *P. regina* positive for urobilinoid signals days after ingestion of dog (*Canis lupus familiaris*) feces. Results are from a controlled laboratory experiment in which flies (N = 4 per time period) were initially exposed to dog feces for 4 h and then killed at 1 d intervals up to 14 d post-exposure (general feeding experiment procedure outlined previously[20]).

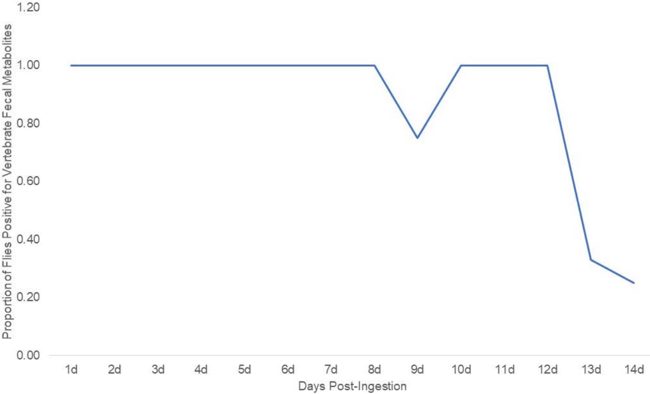
1.20
1.00
0.80
0.60
0.40
0.20
0.00
Proportion of Flies Positive for Vertebrate Fecal Metabolites
1d
2d
3d
4d
5d
6d
7d
8d
9d
10d
11d
12d
13d
14d
Days Post-Ingestion
